# Loop‐mediated isothermal amplification: Development, validation and application of simple and rapid assays for quantitative detection of species of Arcobacteraceae family‐ and species‐specific *Aliarcobacter faecis* and *Aliarcobacter lanthieri*

**DOI:** 10.1111/jam.14926

**Published:** 2020-12-05

**Authors:** I.U.H. Khan, A. Becker, M. Cloutier, M. Plötz, D.R. Lapen, G. Wilkes, E. Topp, A. Abdulmawjood

**Affiliations:** ^1^ Ottawa Research and Development Centre (ORDC) Agriculture and Agri‐Food Canada Ottawa ON Canada; ^2^ Institute of Food Quality and Food Safety Research Center for Emerging Infections and Zoonoses (RIZ) University of Veterinary Medicine Foundation Hannover Germany; ^3^ Natural Resources Canada Ottawa ON Canada; ^4^ London Research and Development Centre, Agriculture and Agri‐Food Canada London ON Canada

**Keywords:** Arcobacteraceae, loop‐mediated isothermal amplification, quantitation, *Aliarcobacter faecis*, *Aliarcobacter lanthieri*

## Abstract

**Aim:**

The family Arcobacteraceae formerly genus *Arcobacter* has recently been reclassified into six genera. Among nine species of the genus *Aliarcobacter*, *Aliarcobacter faecis* and *Aliarcobacter lanthieri* have been identified as emerging pathogens potentially cause health risks to humans and animals. This study was designed to develop/optimize, validate and apply Arcobacteraceae family‐ and two species‐specific (*A. faecis* and *A. lanthieri*) loop‐mediated isothermal amplification (LAMP) assays to rapidly detect and quantify total number of cells in various environmental niches.

**Methods and Results:**

Three sets of LAMP primers were designed from conserved and variable regions of 16S rRNA (family‐specific) and *gyr*B (species‐specific) genes. Optimized Arcobacteraceae family‐specific LAMP assay correctly amplified and detected 24 species, whereas species‐specific LAMP assays detected *A. faecis* and *A. lanthieri* reference strains as well as 91 pure and mixed culture isolates recovered from aquatic and faecal sources. The specificity of LAMP amplification of *A. faecis* and *A. lanthieri* was further confirmed by restriction fragment length polymorphism analysis. Assay sensitivities were tested using variable DNA concentrations extracted from simulated target species cells in an autoclaved agricultural water sample by achieving a minimum detection limit of 10 cells mL^−1^ (10 fg). Direct DNA‐based quantitative detection, from agricultural surface water, identified *A. faecis* (17%) and *A. lanthieri* (1%) at a low frequency compared to family‐level (93%) with the concentration ranging from 2·1 × 10^1^ to 2·2 × 10^5^ cells 100 mL^−1^.

**Conclusions:**

Overall, these three DNA‐based rapid and cost‐effective novel LAMP assays are sensitive and can be completed in less than 40 min. They have potential for on‐site quantitative detection of species of family Arcobacteraceae, *A. faecis* and *A. lanthieri* in food, environmental and clinical matrices.

**Significance and Impact of the Study:**

The newly developed LAMP assays are specific, sensitive, accurate with higher reproducibility that have potential to facilitate in a less equipped lab setting and can help in early quantitative detection and rate of prevalence in environmental niches. The assays can be adopted in the diagnostic labs and epidemiological studies.

## Introduction

The genus *Arcobacter* was first reported in 1991 (Vandamme and De Ley 1991). This Gram negative, nonspore forming, motile and spiral‐shaped bacteria grows well under microaerophilic condition. However, based on genomic and phenotypic analyses, the family Arcobacteraceae (formerly genus *Arcobacter*), within the class *Campylobacteria*, order *Campylobacterales*, has recently been reclassified and divided into six genera including *Arcobacter*, *Aliarcobacter*, *Pseudarcobacter*, *Halarcobacter*, *Malacobacter* and *Poseidonibacter* (Pérez‐Cataluña *et al.* 2018; Oren and Garrity 2019; Pérez‐Cataluña *et al*. 2019). Of these, genus *Aliarcobacter* consists of nine species that have been identified as emerging pathogens causing gastroenteritis, bacteraemia, and sepsis in humans, and mastitis, diarrhoea, abortion and reproductive disorders in animals (Ferreira *et al.* 2016). The epidemiology and pathogenicity of species of family Arcobacteraceae in humans and animals are still not fully understood; therefore, Arcobacteraceae‐associated infections are often considered low risk to public health (Bessede *et al.* 2011). This could be due to misidentification with *Campylobacter* which leads to underestimation of the true cases of Arcobacteraceae‐associated infection. They are ubiquitously prevalent in aquatic environments such as marine and fresh waters, ground water, wastewater and drinking water reservoirs (Jacob *et al.* 1993; Moreira 1998; Rice *et al.* 1999; Moreno *et al.* 2003; Fera *et al.* 2004). Livestock, poultry and wildlife have been considered as main sources of contamination of water (Hausdorf *et al.* 2013) which indicates that water may play vital role in transmission of the disease to humans and animals (Carter *et al.* 1987; Noble and Fuhrman 2001; Wu *et al.* 2011).

Microbial water quality guidelines and standards have been developed for drinking and recreational waters in Canada and other countries where faecal indicator bacteria, including *Escherichia coli* or *Enterococcus* spp., are used as indicators of faecal contamination and prevalence of pathogens in water (Wilkes *et al.* 2009). However, there is weak to no correlation between indicator and pathogens found (Carter *et al.* 1987; Noble and Fuhrman 2001; Wu *et al.* 2011). Collado *et al.* (2008) reported a correlation between *Arcobacter* spp. and faecal indicator in marine and fresh recreational water. However, the culture‐based methods, for such analysis, may require at least 18–96 h to enumerate the number of *E. coli* or enterococci results (Toranzos and McFeters 1997). Moreover the numbers in the water may substantially change in short period of time. Also, the cells may be undetectable and undergo viable but non‐culturable state due to injury and cannot be cultured in the laboratory conditions.

With the evolving technology, genus and species‐specific molecular assays such as conventional and real‐time polymerase chain reaction (PCR) have been developed for detection and enumeration of species of Arcobacteraceae family using 16S rRNA, 23S rRNA, housekeeping, virulence and toxin genes (Bastyns *et al.* 1995; Harmon and Wesley 1996; Houf *et al.* 2000; Whiteduck‐Leveillee *et al.* 2016a, 2016b; Khan *et al.* 2017; Zambri *et al.* 2019; Miltenburg *et al*. 2020). However, these conventional PCR assays are labour and time intensive as well as require post‐PCR agarose gel electrophoresis for confirming the specific amplification reactions. On the other hand, real‐time PCR assay is comparatively rapid for quantitative analysis of pathogens where target amplicons can be observed with the progression of the assay; however, this technique requires more specialized equipment and skilled labour as well as expensive with the fluorescent probes (Liang *et al.* 2009).

The loop‐mediated isothermal amplification (LAMP) has been developed for on‐site specific and rapid detection and identification of target nucleic acid (Notomi *et al.* 2000; Mori *et al*. 2001). The main advantage of the LAMP assay is that it only requires a heating block or water bath to carry out amplification reactions. This technique involves two or three sets of different primers that recognize distinct regions in the target sequence while *Bst* DNA polymerase enhances the amplification reaction, at constant temperature, and produces a large amount of high molecular weight DNA in a short period of time (Niessen *et al.* 2012; Wong *et al.* 2018). However, this technique has some limitations as compared to PCR such as unsuitable for cloning since the amplified product consists of a large DNA chain that makes it less useful. Moreover, it has low success rate than PCR when apply for multiplex assay due to difficulty in the experimental design and procedures (Dhama *et al.* 2014; Sahoo *et al.* 2016).

LAMP assay has been applied as a simple and rapid tool for the detection of various pathogens ranging from bacteria to virus, protozoa and fungi in various food and environmental niches (Liang *et al.* 2009; Niessen *et al.* 2012; Wong *et al.* 2018). Similarly, Wang *et al.* (2014) developed a LAMP assay for detection of *A. butzleri*, *A. cryaerophilus* and *A. skirrowii* and compared them with conventional mono‐ and multiplex PCR assays (Houf *et al.* 2000; Douidah *et al.* 2010). Recently, our lab has developed a conventional multiplex PCR assay for distinctive identification of six species including two (*Aliarcobacter faecis* and *Aliarcobacter lanthieri*) new human‐associated species of genus *Aliarcobacter* (formerly genus *Arcobacter*) (Whiteduck‐Leveillee et al. 2015, 2016a; Khan *et al*. 2017).

Considering the high specificity, sensitivity and rapid application of LAMP assay as a diagnostic tool, this study developed/optimized, validated and applied simple, rapid and sensitive family‐ and species‐specific quantitative LAMP assays for rapid identification of species of Arcobacteraceae family, *A. faecis* and *A. lanthieri* in a pure and mixed culture as well as direct DNA‐based quantitation of total number of cells from complex matrices such as agricultural water samples.

## Materials and methods

### Bacterial reference strains and field cultures

For testing the specificity of primers and optimizing three LAMP protocols for amplification reactions, *A. butzleri* ATCC 49616^T^, *A. faecis* LMG 28519^T^ and *A. lanthieri* LMG 28516^T^ along with six other *Aliarcobacter* spp., and 18 species from genus *Arcobacter*, *Halarcobacter*, *Malacobacter*, *Poseidonibacter* and *Pseudarcobacter* as well as 50 other bacterial reference species and strains were also included as a control in the study (Table 1). For the assessment of specificity of LAMP assays, additionally, *A. butzleri* (*n* = 20), *A. cryaerophilus* (*n* = 10), *A. faecis* (*n* = 10), *A. lanthieri* (*n* = 12), *A. skirrowii* (*n* = 4) and *A. cibarius* (*n* = 5) field strains, previously isolated from various environmental sources in our lab and identified by using multiplex PCR assay, were also included in this study (Khan *et al*. 2017). All bacterial cultures were grown according to their respective selective growth media and incubation conditions (Zambri *et al.* 2019). The DNA of each culture was extracted using a boiling method (Khan *et al.* 2013) by suspending a purified single colony of each reference and field strain as well as putative mixed culture isolates recovered from agricultural water and various faecal sources in 1 × TE (10 mmol L^−1^ Tris‐HCl, 1 mmol L^−1^ EDTA, pH 8·0) buffer. The mixed culture was obtained by scraping an ASIA media plate with a sterile loop and suspended in 1 × TE buffer followed by a 10‐min boiling at 100°C. The tube was centrifuged at 10,000 × ***g*** for one minute, and the supernatant containing DNA was transferred to a sterile tube. The DNA was quantified using Qubit^®^ dsDNA HS Assay kit and Qubit 3.0 fluorometer (Thermo Fisher Scientific, Waltham, MA). The DNA was stored at −20°C for further LAMP assays.

**Table 1:** List of species of Arcobacteraceae family and other bacterial species reference strains used in this study

Sr. #	Species	Source	Strain ID
1	*Aliarcobacter* (*Arcobacter*) *lanthieri*	Pig manure	LMG 28516^T^
2	*Aliarcobacter faecis*	Human waste	LMG 28519^T^
3	*Aliarcobacter butzleri*	Human diarrheic stool	ATCC 49616^T^
4	*Aliarcobacter skirrowii*	Lamb feces	ATCC 51322^T^
5	*Aliarcobacter cryaerophilus*	Bovine aborted fetus	NCTC 11885^T^
6	*Aliarcobacter thereuis*	Organs of aborted porcine	LMG 24486^T^
7	*Aliarcobacter trophiarum*	Feces of fattening pigs	LMG 25534^T^
8	*Aliarcobacter cibarius*	Broiler carcasses	LMG 21996^T^
9	*Aliarcobacter lacus*	Wastewater treatment plant	LMG 29062^T^
10	*Arcobacter nitrofigilis*	Roots	ATCC 33309^T^
11	*Halarcobacter (Arcobacter) bivalviorum*	Shellfish	LMG 26154^T^
12	*H. ebronensis*	Estuarine sediment	LMG 31560^T^
13	*H. anaerophilus*	Mussels	LMG 27922^T^
14	*Poseidonibacter (Arcobacter) lekithochrous*	Larvae of scallop	DSM 100870^T^
15	*Malacobacter* (*Arcobacter*) *halophilus*	Hypersaline lagoon	ATCC BAA‐1022^T^
16	*M. marinus*	Mix seawater, starfish and seaweed	LMG 25770^T^
17	*M. molluscorum*	Mussels and oysters	LMG 25693^T^
18	*M. mytili*	Mussels	LMG 24559^T^
19	*M. canalis*	Wastewater channel	LMG 29148^T^
20	*Pseudarcobacter (Arcobacter) defluvii*	Sewage	LMG 25694^T^
21	*P. ellisii*	Mussels	LMG 26115^T^
22	*P. pacificus*	Seawater surface	LMG 26638^T^
23	*P. caeni*	Wastewater treatment plant	LMG 29151^T^
24	*P. suis*	Pork meat	LMG 26152^T^
25	*P. aquimarinus*	Seawater	LMG 27923^T^
26	*P. cloacae*	Sewage water	LMG 26153^T^
27	*P. venerupis*	Shellfish	LMG 26156^T^
28	*Aeromonas allosaccharophila*	Diseased elvers	ATCC 51208^T^
29	*A. bestiarum*	Infected fish	ATCC 51108^T^
30	*A. caviae*	Epizootic of young guinea pigs	ATCC 15468^T^
31	*A. hydrophila*	Ditch water	ATCC 13444^T^
32	*A. jandaei*	Human faeces	ATCC 49568^T^
33	*A. media*	Marine fish	CDC 0435‐84
34	*A. popoffi*	Drinking water production plant	ATCC BAA‐243^T^
35	*A. salmonicida*	Freshwater	CDC 0434‐84
36	*A. schubertii*	Skin	ATCC 43700^T^
37	*A. sobria*	Sludge	ATCC 35994^T^
38	*A. trota*	Human faeces	ATCC 49658^T^
39	*A. veronii*	Red‐leg frog	ATCC 9071^T^
40	*A. bv. veronii*	Amputation Wound	ATCC 35625^T^
41	*Campylobacter jejuni*	Human faeces	ATCC 33291^T^
42	*C. jejuni*	Human faeces	ATCC 29428^T^
43	*C. jejuni*	Human faeces	ATCC 33291^T^
44	*C. jejuni*	Human faeces	ATCC 33292^T^
45	*C. jejuni* subsp. *doylei*	Human faeces	ATCC 49349^T^
46	*C. coli*	Swine	ATCC 43136^T^
47	*C. coli*	–	ATCC 49941^T^
48	*C. coli*	Marmoset faeces	ATCC 43478^T^
49	*C. lari*	Human faeces	ATCC 43675^T^
50	*C. helveticus*	Cat	ATCC 51210^T^
51	*C. fetus subsp. fetus*	Blood	ATCC 15296^T^
52	*C. hyointestinalis*	Intestine of swine	ATCC 35217^T^
53	*C. lanienae*	–	CCUG 44467^T^
54	*C. upsaliensis*	Dog faeces	ATCC 43954^T^
55	*Escherichia coli* O157:H7	Environmental isolate	–
56	*E. coli*	Canine	ATCC 35218^T^
57	*Enterococcus avium*	Clinical isolate	ATCC 49464^T^
58	*E. casseliflavus*	–	ATCC 700327^T^
59	*E. durans*	Human faeces	ATCC 6056^T^
60	*E. faecalis*	Meat	ATCC 7080^T^
61	*E. faecium*	Human faeces	ATCC 6569^T^
62	*E. gallinarum*	Chicken intestines	ATCC 49573^T^
63	*E. hirae*	–	ATCC 8043^T^
64	*E. saccharolyticus*	Straw bedding	ATCC 43076^T^
65	*Pseudomonas shigelloides*	Environmental isolate	–
66	*Salmonella enterica* subsp. *arizonae*	–	ATC C 13314^T^
67	*S. enterica* subsp. *diarizonae*	–	ATCC 12325^T^
68	*S. enterica* subsp. *houtenae*	–	ATCC 29932^T^
69	*Helicobacter pylori*	Human gastric antrum	NCTC 11637^T^
70	*H. typhlonius*	Human caecum	CCUG 48335^T^
71	*H. ganmani*	Intestines of mice	CCUG 43526^T^
72	*H. marmotae*	Woodchuck liver	CCUG 52419^T^
73	*H. cetorum*	Baluga whale faeces	ATCC BAA‐429^T^
74	*Klebsiella pneumoniae*	Human serotype 3	ATCC 13883^T^
75	*Staphylococcus aureus*	Clinical isolate	ATCC 25923^T^
76	*S. epidermidis*	Clinical isolate	ATCC 12228^T^
77	*Streptococcus pyogenes*	Clinical isolate	ATCC 19615^T^

### LAMP primer design

Initially, three Arcobacteraceae family‐specific primer sets, two outer (F3 and B3), two inner (FIP and BIP) and two Loop (LF1 and LB1) primers, were designed from conserved regions (between V6 and V8) of 16S rRNA gene using Primer Explorer software (Eiken Chemical Co.,Tokyo, Japan). Furthermore, two outer, two inner and one forward loop species‐specific primer sets for each *A. lanthieri* and *A. faecis*, from variable regions of *gyr*B gene were designed. All primer sets were selected based on alignment of all available 16S rRNA and *gyr*B gene sequences of species of family Arcobacteraceae using the MegAlign program (DNASTAR Inc., Madison, WI). The primers were synthesized by Eurofins Genomics GmbH (Ebersberg, Germany). The Arcobacteraceae family‐ and species‐specific target genes and primer sequences are listed in Table 2.

**Table 2:** Arcobacteraceae family‐ and species‐specific oligonucleotide primer sequences designed and used for LAMP assays

Target bacteria	Target gene	Primers	Sequences (5′–3′)	bp
Arcobacteraceae spp.	16S rRNA	Arco‐1‐F3	GCT CGT GTC GTG AGA TG	17
		Arco‐1‐B3	CCG CTT CGA ATG AGT TCA	18
		Arco‐1‐FIP	TCA CCT TCC TCC TAC TTG CGT ACT CGT CGT TAG TTG CTA ACA	42
		Arco‐1‐BIP	AGT TCG GAT TGT AGT CTG CAA CTC ACC GTA GCA TAG CTG ATC T	43
		Arco‐1‐LF	CGT TAG AGT TCT CAG CCG AA	20
		Arco‐1‐LB	CAT GAA GTT GGA ATC GCT AGT AAT C	25
*A. faecis*	*gyr*B	Faecis‐1‐F3	ACA AAA ATA ACA GGT GAT GAT GT	23
		Faecis‐1‐B3	GCT GCC ATT AAA GAT TTT TCC	21
		Faecis‐1‐FIP	TCC CAA TTT TCC TTT TGT TTG TCC TGA GAA GGT TTA ATT GCT GTT GT	47
		Faecis‐1‐BIP	GCT CTT ATG TTA GAC CAA TTG CTC AGC TTT TGC ATG AGT TGG ATT	45
		Faecis‐1‐LF	TTG AGG TTC AGG AAC TTT AAC AGA A	25
*A. lanthieri*	*gyr*B	Lanth‐1‐F3	GCA AAC AAA AGG AAA ATT AGG TAG	24
		Lanth‐1‐B3	ACT TTG ACA ATC TGC AAG TT	20
		Lanth‐1‐FIP	CCA TTA TAG CTT TTG CAT GAA CTG GCC TAT TGC TCA AAA ATT AAC TGG T	49
		Lanth‐1‐BIP	GGC AGC AAG AGG AAG AGA AGC GGA AGT GTT CCA ACA CTC AT	41
		Lanth‐1‐LF	GCT AAA AAG GCA AGA GAA TTA ACT AGA A	28

### Optimization of LAMP assays

Initially, an Arcobacteraceae family‐specific LAMP assay was optimized using a real‐time fluorometer (Genie^®^ II, Optigene, Horsham, UK) system with a 25 *μ*L reaction mixture consisted of: 15 *μ*L Isothermal Master Mix ISO‐001 (Optigene), 2·5 *μ*L 10× primer mix containing 0·2 *μ*mol L^−1^ F3/B3, 0·8 *μ*mol L^−1^ FIP/BIP and 0·4 *μ*mol L^−1^ LF/LB, 2·5 *μ*L nuclease‐free water and 5 *μ*L DNA template. The amplification reaction was run at 65°C for 20 min. followed by a melting process initiated and finished at 98 and 80°C with a 0·05 per s ramp rate. Similarly, LAMP assays for *A. faecis* and *A. lanthieri* were optimized by setting up a 25 *μ*L reaction containing 15 *μ*L Isothermal Master Mix ISO‐001, 5 *μ*L DNA template, 2·5 *μ*L nuclease‐free water and primer mix (0·2 *μ*mol L^−1^ F3/B3, 2·0 *μ*mol L^−1^ FIP/BIP, 1·0 *μ*mol L^−1^ LF/LB) with annealing temperatures 67 and 68·5°C respectively for 20 min followed by melting temperature (Table 3).

**Table 3:** Optimized LAMP primer concentrations and protocols for Arcobacteraceae family‐ and species‐specific detection and identification

Target bacteria	Primer conc. (*μ*mol L^−1^)			Assay conditions	
	F3/B3	FIP/BIP	LF/LB	Annealing	Melt Temp. (°C)
Arcobacteraceae spp.	0·2	0·8	0·4	65°C for 20 min	86
*A. faecis*	0·2	2·0	1·0	67°C for 20 min	81
*A. lanthieri*	0·2	2·0	1·0	68·5°C for 20 min	82

The specificity of *gyr*B gene‐based LAMP amplification reaction was further confirmed by restriction fragment length polymorphism (RFLP) analysis. The specific restriction sites in the LAMP products were analysed and selected with NEB cutter V2.0 (http://tools.neb.com/NEBcutter2). The assay was carried out by selection of restriction enzyme *Alu*I (New England Biolabs GmbH, Frankfurt, Germany) targeting AGCT restriction site located between F1 and B1 primer annealing positions using SeqBuilder Pro (DNASTAR Inc., ver. 15.3, Madison, WI, USA). A 5 *µ*L amplified product was subjected to restriction digestion in 50 *μ*L solution using digestion conditions specified by the manufacturer. The DNA fragments were separated by electrophoresis on 2% agarose gels.

### Assessment of assay specificity, sensitivity and quantitation

To assess the specificity of primers, degree of sensitivity of optimized LAMP protocols and quantitation of total number of cells present in a given field sample, a simulation assay was carried out where known number of cells of *A. butzleri*, *A. faecis* and *A. lanthieri* reference strains were spiked in a highly turbid agricultural water to measure the purity of DNA and detection of least number of cells mL^−1^. For this purpose, initially, the cells of each reference strain were grown in Arcobacter broth media, and incubated at 30°C under microaerophilic (85% N_2_, 10% CO_2_ and 5% O_2_) conditions with continuous shaking at 125 rev min^−1^ for 17 h to obtain exponential growth phase and 0·02 optical density at 420 nm. The cells were collected and resuspended in 1 mL 1 × TE buffer. The cell concentration of each target species was measured on Arcobacter selective isolation agar (ASIA) and incubated under conditions as mentioned above. The known cell concentration (10^7^ cells mL^−1^) of each target species was spiked in an autoclaved agricultural water sample and 10‐fold serially diluted (10^7^–10^1^ cells mL^−1^). Each serially diluted spiked water sample with known cell concentration was filtered through a 0·22 *μ*mol L^−1^ sterile nitrocellulose filter. The autoclaved agriculture water sample was also filtered and used as a negative control. The spiked filters were further processed for DNA extraction using DNeasy PowerSoil kit (Qiagen; formerly MoBio PowerSoil DNA Isolation Kit) according to the manufacturer’s instructions. The purity and concentration of DNA was measured by Qubit 3.0 fluorometer using Qubit^®^ dsDNA HS Assay kit. The extracted DNA was stored at −20°C for further use.

### Validation, application and quantitative analysis of optimized LAMP assays

The optimized LAMP assays were further validated by testing 40 mixed cultures of family Arcobacteraceae species recovered from agricultural water and various faecal samples on ASIA media. For further application of these assays, the spiked water DNA of each target species was added with decreasing equivalent number (from 10^5^ to 10^1^) of cells mL^−1^ corresponding to varying concentrations (ranging from 10 pg to 10 fg) of DNA mL^−1^ to use for quantitation of total number of cells. The specificity and quality of amplified products were confirmed by analysing melting temperatures and compared with melting temperature obtained for *A. butzleri*, *A. faecis* and *A. lanthieri* amplicons. Furthermore, to evaluate and apply the optimized LAMP primers and protocols, 173 agricultural surface water samples were collected from South Nation River, Ontario, Canada. The watershed is mainly dominated by the dairy farming and mix‐uses of cash crops (Wilkes *et al.* 2009). A total of nine sites were selected and surface water samples were collected from May to November in 2017 and 2018. One litre water sample from each site was collected in a sterile polyethylene bottle and delivered to the AAFC‐ORDC lab for microbiological analysis. For the detection and quantitation of total number of species of family Arcobacteraceae (representing species of six newly classified genera), *A. faecis* and *A. lanthieri*, the water samples were processed by membrane filtration using 0·22 *µ*m filters. The filters were further processed for total genomic DNA extraction using DNeasy PowerSoil kit. The DNA was quantified and tested for detection and quantification of total number of cells 100 mL^−1^.

## Results

### Optimization and specificity of LAMP assays

In the first phase of the study, the designed family‐ and species‐specific primers and LAMP protocols were optimized by adjusting optimal annealing temperature (ranging from 60 to 70°C) and tested with the reference species including *A. butzleri*, *A. faecis* and *A. lanthieri*. All primers specifically amplified at their specific annealing (65, 67 and 68·5°C) and melting temperatures (86, 81 and 82°C), respectively (Table 3), by showing typical amplification curves. Furthermore, a total of 17 reference species of five newly classified genera and 61 field isolates were tested for genus‐specificity where all DNA typically amplified at 65°C with the melting curve observed at 86°C. On the other hand, *A. faecis* and *A. lanthieri* species‐specific primers only amplified each target species including reference and field strains by showing amplification of target gene at 67 and 68·5°C and melting curves at 81 and 82°C respectively. All three LAMP assays were further tested for their specificity using Arcobacteraceae closely‐related or other human‐associated bacterial species. Of the total 27 species of family Arcobacteraceae, all 16 species of genus *Arcobacter*, *Aliarcobacter*, *Malacobacter* and *Poseidonibacter* showed positive amplification reactions as compared to genus *Pseudarcobacter* and *Halarcobacter* where among 11 species, all except *H. ebronensis*, *P. caeni* and *P. pacificus* showed positive amplification reaction. Overall, the result indicates that the designed primers and optimized LAMP protocol are highly (89%) specific to all target genera and species of family Arcobacteraceae. In addition, all control DNA templates showed negative amplification reaction to *A. faecis* and *A. lanthieri* species‐specific LAMP assays.

Species‐specific LAMP assays were further confirmed using RFLP analysis of the LAMP amplified products of *A. faecis* and *A. lanthieri*. After using the restriction enzyme *Alu*I, a single restriction site for *A. faecis* LAMP amplified product yielded two (104 and 111 bp) fragments as compared to *A. lanthieri* where three (42, 95 and 108 bp) fragments were obtained (Fig. S1a,b).

### Validation and development of standard curves for quantitative analysis

**Figure 1:**
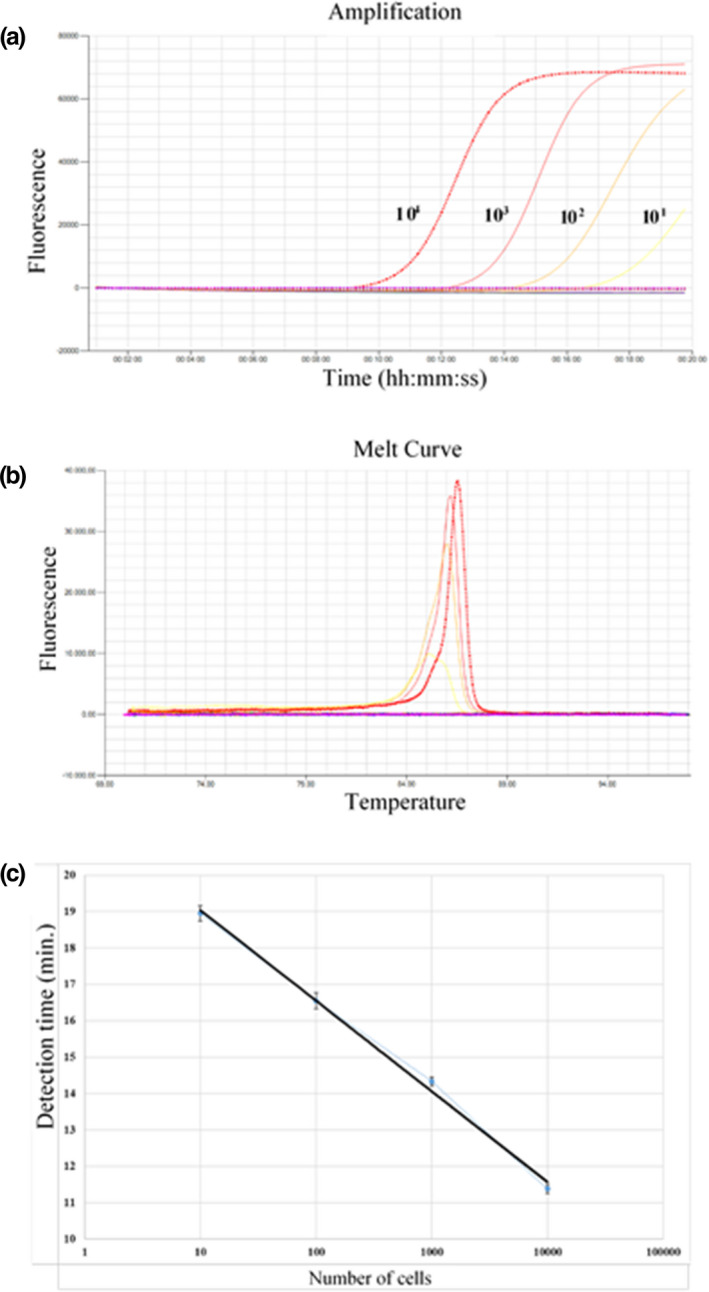
(a) Amplification signals of *A. butzleri* reference strain showing number of cells (ranging from 10^4^ to 10^1^ cells mL^−1^) varying DNA concentrations of a spiked culture in an agricultural water; (b) Annealing curve reactions of different cell numbers of *A. butzleri* reference strain showing a specific relative intensity at 86ºC against negative control; (c) Standard curve for *A. butzleri* using genus‐specific LAMP‐based assay where amplification of 16S rRNA gene‐based genus‐specific LAMP primers targeting *A. butzleri* DNA with an increasing number of cells (ranging from 10^1^ to 10^4^) corresponding to various DNA concentrations (ranging from 10 pg to 10 fg)

The optimized LAMP assays were further validated by testing putative cultures of family Arcobacteraceae recovered from surface water (*n* = 9) and various faecal (*n* = 31) samples. All 40 culture isolates were amplified, yielded typical melting curves and identified to family‐level. Whereas, *A. faecis* was detected in all water and 28 faecal samples compared to *A. lanthieri* where six water and 13 faecal samples were positively amplified with *A. lanthieri* primers. The results also showed that both target species were simultaneously detected in six water and 10 faecal samples indicating the common prevalence of these species in environmental niches. After the optimization of three LAMP assays, family‐ and *A. faecis* and *A. lanthieri* species‐specific standard curves for quantitation of total number of cells were developed using the spiked DNA with the known cell concentration equivalent (ranging from 10^4^ to 10^1^ cell mL^−1^) corresponding to the DNA concentrations (ranging from 10 pg to 10 fg mL^−1^). Based on the fluorescent signals, typical amplification curves at specific melting temperatures were obtained while negative controls did not show a cross‐amplification reaction to the specific LAMP primers and protocols. The least quantification detection limit 10 cells mL^−1^ was achieved (Fig. 1a–c).

### LAMP assay application and direct DNA‐based quantitation of total cells in environmental samples

The optimized newly developed LAMP family‐ and species‐specific assays were further applied for detection and quantification of Arcobacteraceae family‐level as well as *A. faecis* and *A. lanthieri* species in 173 agricultural water samples. Of the total 173 samples, 161 (93%) samples showed typical amplification and melting curves comparable to the family Arcobacteraceae standard where the total number ranged from 57 to 2·5 × 10^5^ cells 100 mL^−1^. The results show that species of family Arcobacteraceae are commonly prevalent in the agricultural water. On the other hand, when DNA was tested for speciation, of the total 161 family‐level positive water samples, 27 (17%) and two (1%) showed typical amplification for *A. faecis* and *A. lanthieri*, respectively, and melting curves similar to the standards while one sample was positively amplified for both target species. The total number of cells of each target species was ranged from 21 to 3·7 × 10^4^ cells 100 mL^−1^. The results showed that the newly developed assays are rapid, robust and simple for detection and quantitative analysis of species of Arcobacteraceae family, *A. faecis* and *A. lanthieri* in various environmental niches.

## Discussion

Conventional culture‐based methods for recovering species of various species of family Arcobacteraceae have limitations including culturing time (4–6 days), potential for false‐positive or negative inference, reduced bacterial viability between collecting and processing samples due to microaerophilic nature, in‐lab environment and growth rate (culturable vs. non‐culturable) that may impact obtaining results in timely‐manner (Roszak and Colwell 1987). Although the nonculturable cells of Gram‐negative bacteria have inability to cause infections, they significantly contribute to the antigen and endotoxin level that may activate immunological response. Therefore, it is important to detect the total number of cells (viable and non‐viable cells) without culturing for rapid screening of environmental, food and clinical samples for pathogen detection. Compared to culture‐based identification including morphology and physico–chemical properties and antibody‐based assay, molecular methods including PCR and real‐time PCR are more reliable and rapid especially when species‐level identification is required. Although, these molecular tools are useful and adopted by diagnostic and analytical labs, they require more than 2–3 h excluding culturing time and DNA preparation. Therefore, LAMP assay has advantages over PCR methods where the target pathogen can be rapidly and accurately detected as well as specifically quantified by accumulation of 10^9^ copies of the target in less than 60 min. Moreover amplification reaction of a target nucleic acid can be performed under isothermal conditions that does not require thermal cycle system and can be conducted in a water bath or heated block (Notomi *et al.* 2000).

Considering the significance of LAMP assay, there was a need to develop a more accurate, reliable and rapid identification system that can detect species of multiple genera that are potentially pathogenic to humans and animals. In recent years, only one LAMP assay has been developed for quantitative detection of *A. butzleri*, *A. cryaerophilus* and *A. skirrowii* (Wang *et al.* 2014). However, this assay was only tested for three species, and with the substantial increase in the number of species over the last few years, none of the other species of family Arcobacteraceae were investigated for the specificity and sensitivity of LAMP primers and protocol.

In this study, we developed species‐specific LAMP assays for *A. faecis* and *A. lanthieri* using variable regions of *gyr*B housekeeping gene, responsible for performing essential bacterial function, whereas conserved regions of gene encoding 16S rRNA was selected for Arcobacteraceae family‐specific primer design. These genes have previously been used for detection of species of family Arcobacteraceae in environmental, clinical and food samples (Douidah *et al.* 2010; Khan *et al.* 2017). Wang *et al.* (2014) developed a LAMP assay to detect species of family Arcobacteraceae in poultry using 23S rRNA as a target gene. To the best of our knowledge, this is the first Arcobacteraceae family‐ and specific‐specific LAMP‐based study where 16S rRNA and *gyr*B genes were selected for designing LAMP primers and a maximum number of species have been tested for optimization, validation of protocols and assessment of specificity and sensitivity of LAMP assays. In order to obtain the best amplification and specificity results, the primers including F3 and B3 were designed with <300 bp DNA fragment size. In addition, loop primers were also designed to accelerate amplification and reduce reaction time (Nagamine *et al.* 2002). Similarly, for specific and sensitive amplification of target gene and species, optimization of LAMP protocol significantly depends on several important factors including primer and DNA template concentration, annealing temperature and type of DNA polymerase used (Notomi *et al.* 2000). Therefore, these parameters were carefully applied in order to determine an optimal protocol that can be used for obtaining maximum amplification reaction in terms of specificity and intensity for rapidly detecting target DNA. Various concentrations for each primer set (ranged from 0·1 to 2·0 *µ*mol L^−1^) were used for assessing the intensity, specificity and reproducibility of the amplification reaction for each targeted species. The obtained amplification for all primer sets used for family‐ and species‐specificity was within the range of primer concentrations tested. In the second step, range of annealing temperatures (from 65 to 70°C) and time (20–25 min) were tested separately for each target for determining suitable temperature and time for specificity with high intensity of amplification reaction. Each developed LAMP assay was able to specifically detect each target DNA with a high intensity at different temperature 65°C (family‐specific), 67°C (*A. faecis*) and 68·5°C (*A. lanthieri*) in less than 40 min including annealing and melting curve steps. The respective results also confirmed that each newly developed and optimized LAMP assay was efficiently producing specific amplification and melting curves for all species, except *H. ebronensis*, *P. caeni* and *P. pacificus*, of six genera of Arcobacteraceae as well as each targeted species. Although the designed primers for species of Arcobacteraceae family showed homogeneity in the nucleotide sequences where the Blast research results showed ≥99% homology to their corresponding species, it is also possible that there is mismatches in the species tested in the study. Therefore, further research is required to identify degree of heterogeneity to strain‐level.

One of the major objectives of this study was to validate the specificity of the optimized LAMP assays by testing other reference and field strains. Therefore, Arcobacteraceae family‐ and species‐specific LAMP assays were applied to a total of 27 species representing six genera along with 61 field isolates including *A. butzleri*, *A. faecis*, *A. lanthieri*, *A. cryaerophilus* and *A. skirrowii* as well as 50 other bacterial reference strains. Each LAMP assay specifically amplified target species and strains and did not show cross‐amplification with other tested control species and strains. The specificity of the assays was further evaluated for the detection to family‐ and species‐level in a mixed putatively positive cultures recovered from selective growth media. The LAMP assays correctly detected all putative mixed cultures to family‐level whereas 48 and 90% cultures were identified as *A. faecis* and *A. lanthieri* respectively. Interestingly, of these, 85% cultures showed positive amplification reaction for both target species which showed incongruence with our previous conventional mPCR study results where only 20% cultures were positive for multiple species (Khan *et al.* 2017). The results of this study indicate that the newly developed LAMP assays are more specific and sensitive to detect multiple species in a mixed culture especially in those cases where one of the target species is present at a low concentration.

After successful optimization and validation of these novel LAMP assays, the major aspect of this study was to assess the degree of sensitivity of these assays for quantification of total number of cells in a complex environmental sample. Initially, we developed a standard curve from a spiked culture of target species in an autoclaved agricultural water where a minimum detection limit of 10 cells mL^‐1^ was achieved. Our limit of detection is similar to the previous study where Notomi *et al.* (2000) described that LAMP assay has an ability to detect less than 10 copies of target sequence. The standard curve was further applied to the field samples for evaluating the sensitivity of the assays where a minimum of ≤10 cells mL^−1^ for Arcobacteraceae family and target species in agricultural water samples was observed compared to a minimum 200 CFU and 2 CFU per reaction in chicken meat and the DNA isolated from cultured bacteria respectively (Wang *et al.* 2014). This difference could be due to the DNA purity and inhibitors that may interfere and reduce the amplification sensitivity of the assay; therefore, various efforts have been made to obtain highly purified nucleic acid from various environmental and clinical niches (Moreira 1998; Bergallo *et al.* 2006). The total number of cells based on quantitative detection to genus‐level were ranged from 57 to 2·5 × 10^5^ cells 100 mL^−1^ and to species‐level for *A. faecis* (ranged from 21 to 3·7 × 10^4^ cells 100 mL^−1^) and *A. lanthieri* (ranged from 45 to 3·2 × 10^2^ cells 100 mL^−1^), respectively. Although the standard curves were developed from the DNA concentrations (10 pg to 10 fg) against the number of cells (10^5^–10^1^ CFU mL^−1^), obtained from the culture‐based data, with 10 cells mL^−1^ as a least detection limit, the amplification reaction of total DNA was observed below 10 cells mL^−1^ which indicates the sensitivity and specificity of the assays and purity of the total DNA that could quantify total number of cells lower than the detection limit in field samples. The infectious dose of genus and species of *Arcobacter* has not been determined yet; however, 10^3^ cells mL^−1^ is considered as an infectious dose when ingested at this concentration (USEPA 2012). Hence, the sensitivity‐level of detection of these assays has shown that the cell concentration is within the range of infectious dose that can potentially cause health risks. In this context, further research efforts are underway to use these newly developed assays in combination with propidium monoazide (PMA) or ethidium monoazide (EMA) to estimate the number of live/dead (viable and non‐viable) cells and compare with culture‐based method for accurate enumeration of cells presents in environmental or clinical samples. Recent study has developed PMA‐based qPCR assay for the detection of viable cells of Arcobacteraceae spp. in shellfish (Salas‐Massó *et al.* 2019). However, the major disadvantages such as toxic effect and penetration of these dyes into live or dead cells can sometime under‐ or overestimate cells (Nocker *et al.* 2006; Taylor *et al.* 2014). Therefore, it requires optimization of dye concentration and exposure time without impacting on the specificity and sensitivity of these newly developed LAMP assays.

In conclusion, the results of three simple and rapid newly developed LAMP assays showed that assays are more specific, sensitive, accurate and have higher reproducibility than previously developed family‐ and species‐specific molecular assays. Moreover, the study results provide sufficient data that would support in adopting these novel LAMP assays that have potential to facilitate on‐site or in a less equipped lab setting for rapid and direct DNA‐based quantitative screening of environmental and clinical samples for *A. faecis* and *A. lanthieri*. In compared to our previous real‐time PCR assays (Miltenburg *et al*. 2020), the major advantages of these LAMP assays are that they do not require post‐PCR analysis and can be completed in less than 40 min with a minimum to no risk of cross‐reaction with other species of closely related genera. These assays can also contribute to improve our ability and understanding on the early quantitative detection and identification, rate of prevalence and ecology of these emerging pathogens in environmental niches as well as can be applied in the diagnostic labs and epidemiological research studies.

## Conflict of Interest

The authors have declared no conflict of interest.

## Authors’ contribution

IK and AA conceived research project, performed sequence analyses and designed experiments. IK, AA and AB designed LAMP primers and protocols, conducted experiments, analysed data and drafted manuscript. MM, MP, DL, GW and ET coordinated in identifying sampling sites and sample collection procedure as well as contributed in data interpretation and editing of manuscript. All authors reviewed, edited and approved the final manuscript.

## Supporting information

**Figure S1.** RFLP analysis of LAMP species‐specific amplified products using *Alu*I restriction enzyme.
