# Supplementary figures and images for: Loop‐mediated isothermal amplification: Development, validation and application of simple and rapid assays for quantitative detection of species of Arcobacteraceae family‐ and species‐specific Aliarcobacter faecis and Aliarcobacter lanthieri
Source: J Appl Microbiol. 2020 Dec 5;131(1):288–99. doi: 10.1111/jam.14926 (PMC8359143; doi:10.1111/jam.14926)

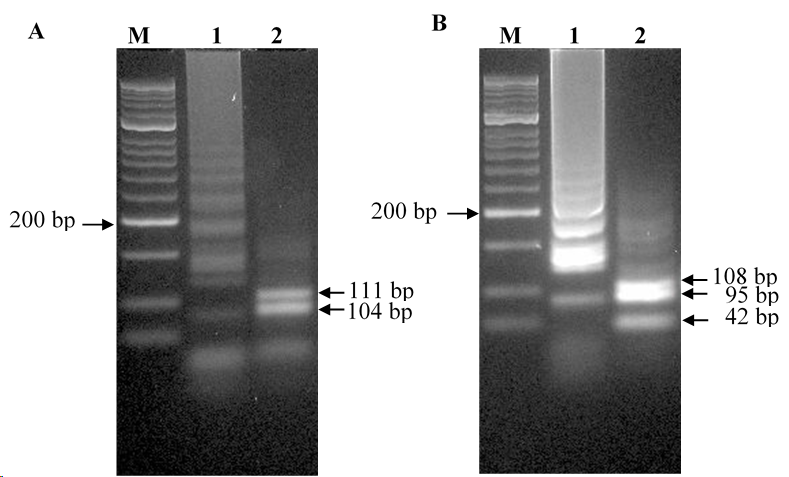

Supplement: Supplementary file 1 — Figure S1. RFLP analysis of LAMP species‐specific amplified products using AluI restriction enzyme. [file JAM-131-288-s001.tif]
